# Circular RNA Involved in the Protective Effect of *Malva sylvestris* L. on Myocardial Ischemic/Re-Perfused Injury

**DOI:** 10.3389/fphar.2020.520486

**Published:** 2020-09-25

**Authors:** Yongzhi Xiao, Diafara Boureima Oumarou, Shuang Wang, Yingzhe Liu

**Affiliations:** ^1^ Department of Ultrasonography, The Second Xiangya Hospital, Central South University, Changsha, China; ^2^ Department of Anesthesiology, The Second Xiangya Hospital, Central South University, Changsha, China; ^3^ Department of Medical Research Center and Clinical Laboratory, Xiangya Hospital, Central South University, Changsha, China; ^4^ Xiangya International Medical Center, National Clinical Research Center for Geriatric Disorders, Xiangya Hospital, Central South University, Changsha, China

**Keywords:** ischemic heart disease, *Malva sylvestris* L., traditional medicine, inflammasome, circular RNA

## Abstract

Ischemic heart disease has become a major health challenge worldwide. Malva sylvestris L. (MS) is a traditional herbal medicine with anti-inflammatory properties and have been used as antioxidant and anti- inflammatory agent in infectious diseases and inflammatory diseases.In this study, we aimed at elucidating the mechanism of MS against ischemia-reperfusion (I/R)–induced injury in vivo and in vitro. The I/R animal model in rats and oxygen glucose deprivation/re-oxygenation (OGD/Re) model in H9c2 cells were used in this study. MS was used to pre-treat the rats and cells. Electrocardiogram, histology staining, qPCR, ELISA, CCK-8, and circRNA microarray were performed. We found that pre-treatment with MS extract attenuate OGD/Re-induced cell apoptosis and cell viability inhibition in H9c2 cells. In addition, pre-treatment with MS protected against I/R injury in vivo. The protective effects of MS pre-treatment were associated with inflammatory genes expression and cytokines release. Further mechanistic investigation revealed that MS protected cardiomyocytes through regulating circular RNA (circRNA). We identified a novel circRNA circ003593 that mediated the protective role of MS in vitro through NLRP3 complex, which was associated with reperfusion injury salvage kinase (RISK) signaling pathway. Conclusion: this study is the first time to demonstrate the protective role of MS on I/R injury. Our findings reveal a novel circRNA circ003593-mediated the protective role of MS through NLRP3 inflammasome. Circ003593 may serve as a potential therapeutic target for ischemic heart diseases.

## Introduction

Ischemic heart disease (IHD) has high mortality and morbidity rates (Lee et al., 2016). When IHD occurred, cardiac tissues are damaged by the toxic effects of reactive oxygen species, the release of apoptogenic factors from mitochondria, and free radicals generated by infiltrated activated immune cells during hypoxia (Frangogiannis, 2014; Lesnefsky et al., 2017). Many traditional Chinese medicines have been used for treating IHD (Wang D. et al., 2016).

*Malva sylvestris* L. (MS) is a traditional herbal medicine that contain essential oils, vitamins C, fatty acids, and various sterols, which have anti-inflammatory properties and have been used as antioxidant and anti- inflammatory agent in infectious diseases and inflammatory diseases (Veshkurova et al., 2006; Hicsonmez et al., 2009; Usami et al., 2013; Martins et al., 2014; Elsagh et al., 2015; Jabri et al., 2017). However, the mechnisms underlying MS protectes I/R injury are not fully clear. In this study, we aimed to elucidate the anti-apoptosis and anti-inflammation mechanism of MS againstischemia-reperfusion (I/R) injury in vivo and in vitro.

## Methods and Materials

### Preparation of *Malva Sylvestris* L.

The authenticated Malva sylvestris L. were collected from botanical garden (Xuzhou, China). The air-dried MS plants were coarsely ground into leaf powder, and the powder was macerated in 95% methanol for seven days. The concentrate was sifted through and the filtrate was dried utilizing a rotating evaporator. The final extract was used for next experiments at the dose of 300 mg/kg (in vivo) or 6 µg/mL (in vitro). 

### Animal Experiment Designs

Sprague Dawley (SD) rats (n = 20; male, 8 weeks old, 200–250 g) were randomly classified into four groups [sham + normal saline (NS), n = 5; sham+ MS, n = 5; I/R + NS, n = 5; IR+MS, n = 5]. The I/R rats were established by ligating the left anterior descending coronary artery (LAD) for 45 min and reperfusion for 24 h; the sham group received the same operation without ligation. Twelve hours before the operation, the rats were intraperitoneally administrated with 300 mg/kg of MS or the equal volume normal saline. Animal experiments were approved by the Ethical Committee for Animal Research of Central South University.

### Electrocardiogram (ECG) Recording

ECG heartbeats monitor (BL-420S biological function experimental system, Chengdu Taifeng Technology Co., Ltd., Chengdu, China) is utilized to observe and record a standard limb II ECG before and during myocardial ischemia and reperfusion under anesthesia. The ST changes were assessed during 30 min period of ischemia, and the first 10 min of reperfusion.

### Hematoxylin Eosin (H&E) Staining

The hearts were removed for H&E staining according to previously described (Tian et al., 2017). Briefly, the paraffin embedded tissues were cut into four-µm slides and stained followed the commercial H&E staining kit (ab245880, Abacm). The slides were stained with hematoxylin for 3 minutes and then stained with Eosin Y for 3 minutes. Finally, the slides were viewed by using a microscope. The sizes of the infiltrating area of the inflammatory cells (mainly neutrophils, lymphocytes, and monocytes) were calculated in infarcted region on eight sections per animal at 40× magnification by using the Image-Pro Plus software. The average area was normalized by sham + NS group. The final data was expressed as relative inflammatory cell infiltration area.

### Triphenyltetrazolium Chloride (TTC) Staining

After anesthetization with pentobarbital sodium, hearts were rapidly harvested and dipped/washed in potassium chloride saline solution buffer. Frozen heart tissues were cut into 2 mm transverse slices and then incubated in 1.5 % TTC (T8877, Sigma) at 37°C for 20 min and fixed with 10 % formalin overnight. Slices image were obtained to calculate the infarct area by ImageJ. The infarcted size was calculated as: size of infarcted region/size of whole heart *100%.

### ELISA for Methylene Dioxyamphetamine (MDA), Superoxide Dismutase (SOD), and Catalase (CAT)

MDA, SOD, and CAT were measured by using an ELISA kit from Nanjing Jiancheng Company (catalog nos.: AOO3 for SOD, A007-2 for CAT, and A003-1 for MDA, Nanjing, China) according to the manufacturer’s protocol.

### Real-Time Quantitative Polymerase Chain Reaction (qPCR)

Animals were sacrificed, and the infarcted heart tissues were obtained 1 day after ischemia reperfusion. The total RNA was isolated from the ischemic heart tissues or cells by using Trizol following the manufacturer’s protocols, and the concentration of RNA was detected by Nano Drop 2000. qPCR for mRNAs and circRNA was measured by SYBR Green Realtime PCR Master Mix (code no. QPK-201, TOYOBO, Japan) following the manufacturer’s recommended protocol on a CFX96 Real-Time PCR Detection System (Bio-Rad, Hercules, CA, USA). Primers sequences were as follow: TNF-α (forward primer, TGGCCTCCCTCTCATCAGTT; reverse primer, ACAAGGTACAACCCATCGGC), IL-1β (forward prime, GCAGTGGTTCGAGGCCTAAT; reverse primer, GCTGCTTCAGACACTTGCAC), IL-6 (forward primer, GTGGCTAAGGACCAAGACCA; reverse primer, ATAACGCACTAGGTTTGCCGA), IL-10 (forward primer, CCCCTTCACTTTCAGGGTCG; reverse primer, GGGGGTTTCTTAGGGGTCAG), circ_003593 (forward primer, GGCCTTGTCTCAAATCAGCT; reverse primer, TCCCCTTTAGTCACCTCAGAG), and GAPDH, (forward primer, CCTCTCATGCACCACCATCA; reverse primer, GCATTGCACCTCAGGGAAGA).

### Cell Culture and Oxygen Glucose Deprivation/Re-Oxygenation (OGD/Re) Model

The rat cardiomyocyte-like H9c2 line was cultured in DMEM medium (11-995-040, Gibco) supplemented with 10% (v/v) fetal bovine serum (FBS) (SH30071.03, Hyclone), 1% penicillin/streptomycin (v/v), (15-140-122, Gibco) and 2 mM L-glutamine (25-030-081, Gibco). The cells were cultured in a humidied under 5% CO2 and 95% N2 at 37°C in a standard incubator and reached about 80% for experiments.

The H9c2 cardiomyocytes were pre-treated with of MS extract (6.0 µg/mL) for 24 h and then for OGD/Re (Zheng et al., 2017). Oxygen glucose deprivation condition was created by incubating the cells in glucose-free medium and an airtight Plexiglas chamber with an atmospheric composition of 5% CO_2_ and 95% N_2_ at 37°C for 3 h. After exposure to oxygen glucose deprivation for 3 h, the cells were reoxygenated by incubating in a standard 5% CO_2_ incubator for 3 h.

### Cell Transfection

To knockdown circ_003593, circ_003593 shRNA (shRNA sequence, CCGGTCTGACGTGGATATTAGTAATCTCGAGATTACTAATATCCAC GTCAGATTTTTG) was constructed and packaged into lentivirus by Genomedi tech Co. Ltd. (Genomedi tech, Shanghai, China). The lentivirus with scramble sequence was used as negative control. Briefly, 1 × 10^6^ H9c2 cells were seeded in six-well plates to an 80% confluence and then infected with lentivirus at 100 multiplicity of infection for 48 h.

### CCK-8 Assay

The cellular viability of H9c2 cells was determined with the CCK-8 assay. The treated cells were seeded into 96-well plates (3000 cells/well), and cell proliferation was assessed using a CCK-8 kit (Beyotime Biotechnology, Shanghai, China) according to the manufacturer’s instructions.

### Annexin V and Propidium Iodide Staining for Apoptosis

The percentage of apoptotic cells were measured by flow cytometry using an Annexin V Alexa Fluor (488)/ propidium iodide (PI) apoptosis kit (V13245, Invitrogen). The cells were washed with cold PBS buffer for twice and incubated with 5 µl of Alexa Fluor 488-annexin V and 1 µl of PI working solution at room temperature for 15 minutes. The celluar fluorescence was analyzed by using a BD FACS CaliburTM flow cytometer.

### CircRNA Microarray

The infarcted heart tissues were used for microarray assay to determine differentially expressed circRNAs using the circRNAs chip (Arraystar circRNAs chip, AraryStar) containing 14,236 probes specific for circular RNAs splicing sites. The microarray hybridization including purifying RNA transcribing into fluorescent cRNA was performed based on the manufacturer’s standard protocols and then hybridized onto circRNA arrays. After the hybridized slides were washed and fixed, the slides were scanned using Agilent Scanner G2505C, followed by the data collection by Agilent Feature Extraction software.

### Annotation and Function Prediction

ccording to the qPCR results, rat_circRNA_003593 (circ003593) was selected for annotation and function prediction. In addition, cytoscape (http://www.cytoscape.org/) was utilized to establish a circRNA-miRNA-mRNA interaction network of circ003593. Functional annotation of genes in the networks was performed using KEGG and GO pathway analysis.

### Immunohistochemistry Staining

The paraffin-embedded tissues were serially cut at 4 μm. Slides were regularly deparaffinized and dehydrated. Tissues were retrieved in boiling citric acid buffer (pH 6.0) for 15 min. After natural cooling, the slices were blocked with normal goat serum and incubated with primary antibody (rabbit polyclonal anti-cleaved caspase 3; 1:200 dilution, catalog no. 2302, Abcam) overnight at 4°C. Following secondary antibody incubation, the sections were stained by using the DAB Detection Kit (Maxim, Xiamen, China). Finally, the sections were counterstained with hematoxylin. Semi-quantification was analyzed using ImageJ.

### Western Blot

Proteins were extracted using RIPA buffer. A BCA Protein Assay kit (Thermo Fisher Scientific, Rockford, IL, USA) were used to determine protein concentrations. Protein (60 μg) was separated on a 10% SDS-PAGE gel and transferred onto polyvinylidene difluoride (PVDF) membranes (Millipore, Billerica, MA, USA). Then, the membranes were blocked in 10% nonfat milk for 1 h and then incubated with corresponding primary antibodies (anti–TNF-α: 1:1,000 dilution, catalog no. ab1793, Abcam; anti–IL-1β: 1:1,000 dilution, catalog no. ab2105, Abcam; anti–IL-6: 1:1,000 dilution, catalog no. ab6672, Abcam; IL-10: 1:1,000 dilution, catalog no. ab34843, Abcam; anti-NLRP3: 1:1,000 dilution, catalog no. ab263899, Abcam; anti-ASC: 1:1,000 dilution, catalog no. ab155970, Abcam; anti-cleaved caspase 1: 1:1,000 dilution, catalog no. ab207802, Abcam; anti–p-AKT: 1:1,000 dilution, catalog no. ab38499, Abcam; anti-AKT: 1:1,000 dilution, catalog no. ab18785, Abcam; anti–p-ERK1/2: 1:1,000 dilution, catalog no. ab214342, Abcam; anti-ERK1/2: 1:1,000 dilution, catalog no. ab17942, Abcam; anti-GAPDH: 1:1,000 dilution, catalog no. ab181602, Abcam) overnight at 4°C. Following incubation with appropriate secondary antibody, the bands were visualized using an ECL kit (Millipore). Signals were quantified by ImageJ software and normalized to GAPDH.

### Statistical Analyses

All the experiments were repeated independently three times. Values are expressed as means ± standard deviation.Statistical analysis was performed using SPSS15.0(SPSS, Inc., Chicago, IL, United States). Student t test and one-way ANOVA followed by the Newman–Keuls test were used for statistical analysis. Statistical significance was determined as p < 0.05.

## Results

### Protective Effect of MS Against OGD/Re-Induced Injury in H9c2 Cells

**Figure 1:**
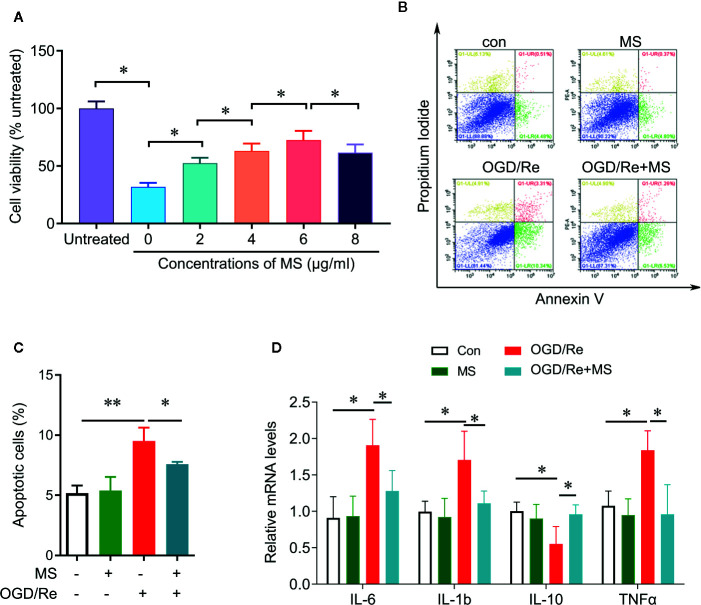
Protective effect of *Malva sylvestris L.* against OGD/Re-induced injury on H9c2 cells. **(A)** CCK-8 was performed to determine the cell viability after indicated treatment in H9c2 cells. **(B**, **C)** Flow cytometry was performed to determine the apoptotic cell after indicated treatment in H9c2 cells **(B)**, and quantification **(C)**. **(D)** qPCR was performed to determine the mRNA expression of IL-6, TNF-α, IL-1β, and IL-10 after indicated treatment in H9c2 cells. *p < 0.05 and **p < 0.01. MS, *Malva sylvestris L.*; OGD/Re, oxygen glucose deprivation/reoxygenation.

To determine whether MS pre-treatment was able to protect against cardiac injury induced by OGD/Re, we examined the cell viability and cell apoptosis after MS pre-treatment and OGD/Re stimulation. H9c2 cardiomyocytes were pretreated with a serial concentrations of MS extracts for 24 h and then were exposed to oxygen glucose deprivation for 3 h followed by reoxygenation for 3 h. Cell viability was then detected by CCK-8 assay. OGD/Re significantly decreases the cell viability compared with control group. Pre-treatment with MS extracts increased the cell viability in OGD/Re-challenged cells at concentrations of 2, 4, 6, 8 µg/mL. MS extract exhibited the highest protective effect at a concentration of 6 µg/mL, whereas higher concentrations provoked a further decline in cell viability (**Figure 1A**). Therefore, we chose the concentration of 6 µg/mL for further analysis. In addition, pre-treatment with MS extracts reduced the apoptotic cells that induced by OGD/Re treatment (**Figures 1B, C**). Further experiments showed that OGD/Re stimulation significantly increased IL-6, IL-1β, and TNF-α expression and decreased IL-10 expression compared with control group. Interestingly, MS pretreatment significantly reversed the effects of OGD/Re on IL-6, IL-1β, TNF-α, and IL-10 expression. These results suggest that pre-treatment with MS extract attenuates OGD/Re-induced cell apoptosis and cell viability inhibition in the H9c2 cells, which is associated with cytokines release.

### Effect of MS Pre-Treatment on Myocardial Infarction

**Figure 2:**
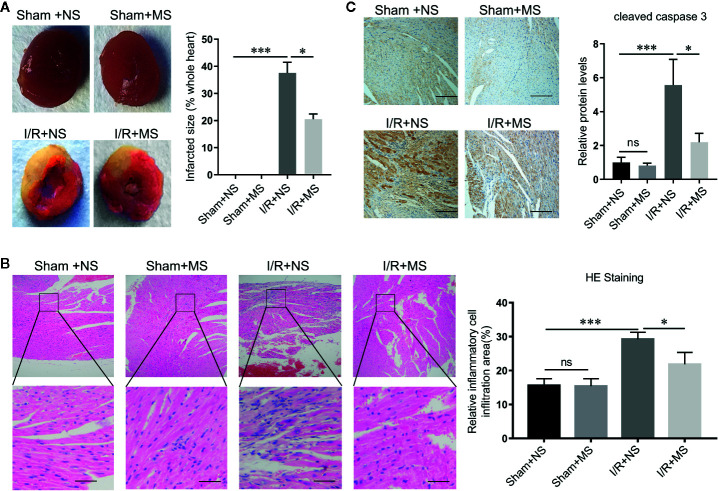
*Malva sylvestris L.* pretreatment decreases the infarcted size. **(A)** TTC staining was performed to determine the myocardial infarcted size in I/R rats with or without MS pre-treatment (N = 5). **(B)** H&E staining was performed to determine the myocardial morphology and inflammatory cell infiltration in I/R rats with or without MS pre-treatment (left), and the area of inflammatory cell infiltration was quantified (right). **(C)** immunohistochemistry staining was performed to determine the expression of cleaved caspase 3 in myocardial infarcted tissues. *p < 0.05 and ***p < 0.001. ns, no significance; MS, *Malva sylvestris L.*; NS, normal saline; I/R, ischemia-reperfusion.

**Figure 3:**
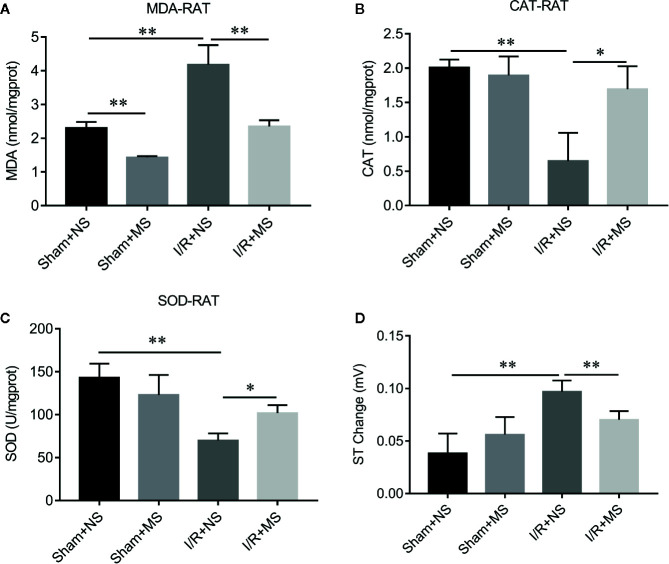
The effects of *Malva sylvestris* L. on MDA, CAT, and SOD in I/R rat model. **(A**–**C)** ELISA was performed to determine the concentration of MDA **(A)**, CAT **(B)**, and SOD **(C)** in I/R rat model with or without MS pre-treatment. **(D)** Electrocardiogram (ECG) records the ST changes in I/R rat model with or without MS pre-treatment. *p < 0.05 and **p < 0.01. MS, *Malva sylvestris* L.; NS, normal saline; I/R, ischemia-reperfusion.

In order to evaluate the effect of MS on myocardial infarction in vivo, we established the ischemia/reperfusion (I/R) animal model. I/R group showed larger myocardial infarcted size than the sham group. Pre-treating with MS (300 mg/kg) significantly decreased the infarcted size compared with I/R group (**Figure 2A**). H&E staining showed a well-arranged myocardial structure (very small intercellular space, and edema does not appear between cells) in the sham group. However, I/R altered muscle fibers morphology with severe myofibrillar interstitial edema and larger inflammatory cells infiltration area (**Figure 2B**). MS pretreatment maintained the myocardium tissue with less irregularly arranged fibers, fewer degenerated cardiac cells and myofiber loss, and smaller inflammatory cells infiltration area than I/R group (**Figure 2B**). In addition, MS pretreatment decreased the expression of cleaved caspase 3, which was induced by I/R (**Figure 2C**). Furthermore, I/R treatment significantly increased the concentration levels of MDA and decreased the activities of CAT and SOD. MS pretreatment significantly reduced the level of MDA and increased the levels of CAT and SOD compared with I/R group (**Figures 3A–C**). MS pre-treatment also significantly reduced I/R-mediated ST elevation (**Figure 3D** and **Supplementary Figure 1**).

**Figure 4:**
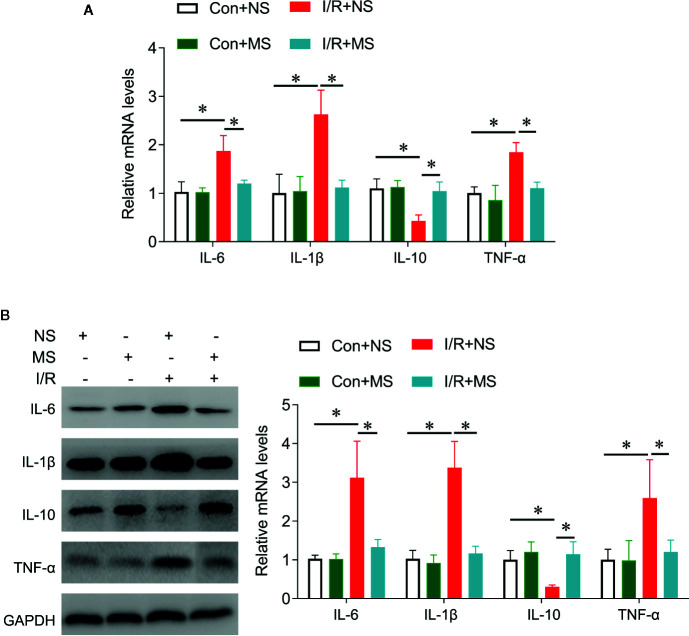
The effects of *Malva sylvestris* L. on inflammatory genes in I/R rat model. **(A)** qPCR was performed to determine the expression of IL-6, IL-1β, IL-10, and TNF-α in the infarcted heart tissues from I/R rat with or without MS pre-treatment. **(B)** Western blot was performed to determine the expression of IL-6, IL-1β, IL-10, and TNF-α in the infarcted heart tissues from I/R rat with or without MS pre-treatment. *p < 0.05. MS, *Malva sylvestris* L.; NS, normal saline; I/R, ischemia-reperfusion.

We next investigated the effects of MS on cytokines release in vivo. I/R significantly increased IL-6, IL-1β, TNF-α expression and reduced IL-10 expression at protein and mRNA levels compared with the Sham group. MS pretreatment significantly decreased the expression of IL-6, IL-1β, TNF-α, while increased IL-10 expression in comparison with I/R groups (**Figure 4**).

### Effect of MS on the Differentially Expressed circRNAs

**Figure 5:**
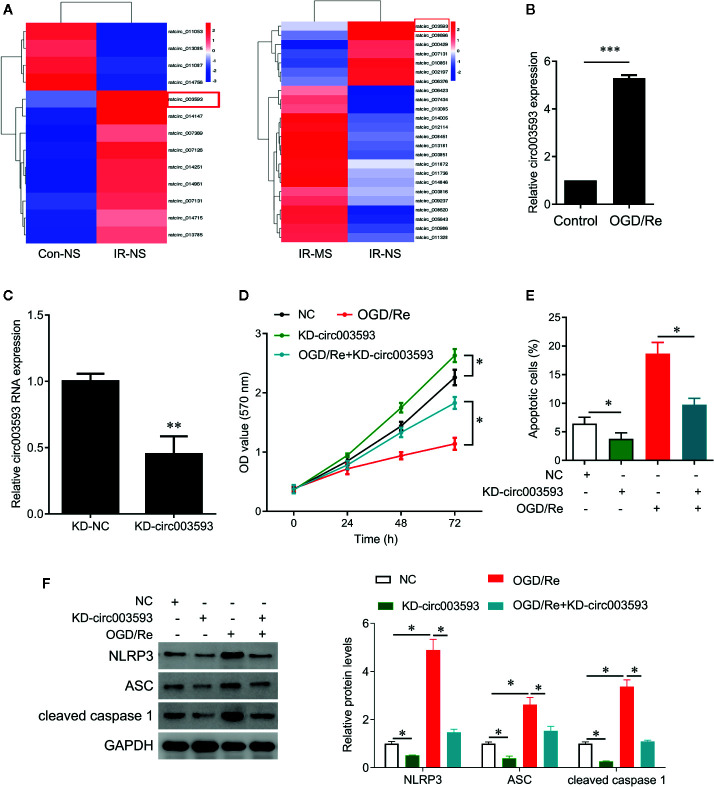
The effects of *Malva sylvestris* L. on circular RNA expression. **(A)** cirRNA expression profiles in I/R rat model with or without MS pre-treatment. **(B)** qPCR was performed to measure the expression of circ_003593 in H9c2 cells after OGD/Re treatment. **(C)** circ_003593 was knocked down by shRNA transfection. **(D)** CCK-8 was performed to determine the cell viability in H9c2 cells after indicated treatment. **(E)** Flow cytometry was performed to determine the apoptotic cell in H9c2 cells after indicated treatment. **(F)** Western blot was performed to determine the expression of NLRP3, ASC, and cleaved caspase 1 in H9c2 cells after indicated treatment. *p < 0.05, **p < 0.01, and ***p < 0.001. MS, *Malva sylvestris* L.; NS, normal saline; I/R, ischemia-reperfusion; KD, knockdown; OGD/Re, oxygen glucose deprivation/reoxygenation; ASC, adapter apoptosis-associated speck-like protein containing a C-terminal caspase recruitment domain.

To investigate the potential mechanism underlying MS protects cardiomyocytes from I/R injury, we obtained circRNA expression profile in myocardial infarcted tissues by circRNA microarray (**Figure 5A**). Thirteen circRNAs were differentially expressed in myocardial infarcted tissues and normal tissues (P < 0.05). Among these aberrantly expressed circRNAs, nine circRNAs were upregulated, and four circRNAs were downregulated by I/R injury (**Figure 5A**). In addition, MS pre-treatment also induced the differentially expressed circRNAs in I/R rats. Among these aberrantly expressed circRNAs, 17 circRNAs were upregulated, and 7 circRNAs were downregulated by MS-pretreatment (**Figure 5A**). We noted that rat_circ_003593 (circ003593) was upregulated by I/R injury and downregulated by MS pre-treatment (**Figure 5A**). We also observed that OGD/Re treatment significantly upregulated the expression of circ003593 in H9c2cells (**Figure 5B**). Thus, we selected circ003593 for further analysis. We knocked down cir003593 in H9c2 cells (**Figure 5C**) and found that cir003593 knockdown significantly rescued OGD/Re-mediated cell proliferation inhibition (**Figure 5D**) and cell apoptosis promotion (**Figure 5E**). In addition, cir003593 knockdown inactivated NLRP3 complex evaluating by the decreased expression of NLRP3, ASC, and cleaved caspase 1. Furthermore, cir003593 knockdown inhibited OGD/Re-mediated activation of NLRP3 complex (**Figure 5F**).

### The miRNA-mRNA Network Targeted by circ003593

**Figure 6:**
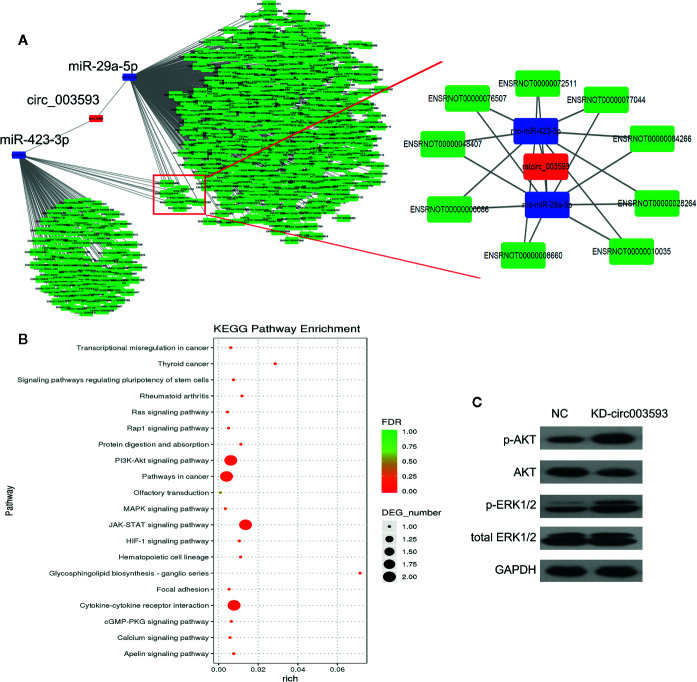
The downstream targets and pathways of circ_003593. **(A)** The predicted miRNA and mRNA targets of circ_003593. **(B)** KEGG pathway enrichment involved in circ_003593. **(C)** Western blot was performed to determine the expression of AKT and ERK1/2 in H9c2 cells after circ_003593 knockdown. KD, knockdown.

The circRNA-miRNA-mRNA interaction network of circ003593 was analyzed using cytoscape software. Nine miRNAs (miR-1199-3p, miR-149-5p, miR-217-3p, miR-22-5p, miR-29a-5p, miR-3550, miR-3568, miR-3575, and miR-423-3p) have binding sites with circ003593. Previous studies have showed that miR-29a-5p and miR-423-3p play important role in I/R injury (Ning et al., 2017; Rizzacasa et al., 2019). Our analysis found that circ003593 interacted with both miR-29a-5p and miR-423-3p. These two miRNAs have nine common target genes (**Figure 6A**). The details of the molecular interactions between miRNA and its targets are described in **Supplementary Table 1**. We hypothesized that circ003593 could regulate the activity of targeted miRNAs as miRNA sponges to modulate circRNA-miRNA-mRNA network. The KEGG pathway enrichment analysis demonstrated that there are four signaling pathways, including phosphatidylinositol-3-kinase (PI3K)-AKT signaling pathway, pathway in cancer, Janus kinase (JAK)-signal transducer and activator of transcription (STAT) signaling pathway, cytokine-cytokine receptor interaction, were highly associated to the predicted targets of circ003593 (**Figure 6B**). In addition, circ003593 knockdown significantly increased the expression of phosphorylated AKT and extracellular signal–regulated kinase 1/2 (ERK1/2), suggesting that circ003593 knockdown activates the reperfusion injury salvage kinase (RISK) signaling pathway (**Figure 6C**).

## Discussion

MS possesses anti-inflammatory and antioxidant activities to protect against myocardial ischemia reperfusion (Prudente et al., 2017). In this study, MS pretreatment reduced myocardial tissue damage and improved favorable morphological changes in the cardiac tissue during I/R injury. Infiltrating leukocytes in infarcted tissues release oxygen free radicals to participate in the pathogenesis of reperfusion injury (Granger and Kvietys, 2015; Yu et al., 2018). I/R-induced myocardial injury decreases the concentration of SOD and CAT but rises the level of MDA. MS pre-treatment inhibited I/R-induced myocardial injury by increasing the concentration of SOD, CAT, and reducing MDA. As far as we know, MDA is useful as an oxidative stress marker, and SOD and CAT represent antioxidant enzymes. These data support that MS ameliorate I/R-induced myocardial injury through anti-oxidative function.

During the response to I/R-induced injury, the injured myocardium are associated with an inflammatory response characterized by the accumulation of leukocytes (Yan et al., 2013); these leukocytes release amounts of cytokines, such as IL-6, IL-1β, and TNF-α, to promote left ventricular dysfunction adverse reactions and heart failure (Oliveira et al., 2018; Yu et al., 2019). Pretreatment with MS significantly decreased the expression of IL-6, IL-1β, and TNF-α and increased the level of IL-10 in cardiac tissues. IL-10 play an important role on anti-inflammatory response by ceasing the pro-inflammatory cascade (Frangogiannis et al., 2000). These observations suggest that MS has an anti-inflammatory activity.

We further investigated the possible mechanism underlying MS protects myocardial. Circular RNAs (circRNA) have been implicated on the effects of traditional medicine (Guo et al., 2017; Altesha et al., 2019). Wang et al found that circRNA MFACR (mitochondrial fission and apoptosis-related circRNA) regulates cardiomyocyte apoptosis by directly targeting miR-652-3p (Wang et al., 2017); and circRNA HRCR inhibits failure by inhibiting miR-223 activity (Wang K. et al., 2016). CircRNA Cdr1as overexpression promotes cardiomyocyte apoptosis by sponging miR-7a (Geng et al., 2016). Recently, Huang found that circRNA circNfix promotes cardiac regenerative repair and functional recovery by inhibiting Y-box binding protein 1 ubiquitin-dependent degradation (Huang et al., 2019). In this study, we identified a novel circRNA, circ003593, which was associated with MS effects on cardiomyocyte proliferation and apoptosis. In addition, circ003593 inactivated the NLRP3 complex, which is the best characterized inflammasome complex and has been linked with various human autoinflammatory and autoimmune diseases (Jo et al., 2016). Activation of NLRP3 leads to recruitment of the adapter apoptosis-associated speck-like protein containing a C-terminal caspase recruitment domain (ASC), resulting in the activation of pro-caspase-1 into its cleaved form. Caspase-1 is known as an inflammatory caspase that plays a role in the maturation of IL-1β into active cytokines and the initiation of pyroptosis by autocatalysis and activation (Lamkanfi, 2011).

We also found that circ003593 might potentially interact with nine miRNAs including miR-1199-3p, miR-149-5p, miR-217-3p, miR-22-5p, miR-29a-5p, miR-3550, miR-3568, miR-3575, and miR-423-3p. Moreover, KEGG pathway analysis indicated that circ_003593 was involved in the pathways of PI3K-AKT signaling pathway, pathway in cancer, JAK-STAT signaling pathway, and cytokine-cytokine receptor interaction. These pathways are closely associated with I/R injury (Negoro et al., 2000; Barry et al., 2007; Frangogiannis, 2012; Yao et al., 2014; Thackeray et al., 2015; Mahajan et al., 2018). We also confirmed that circ003593 knockdown significantly activated PI3K-AKT signaling pathway and ERK1/2 pathway, both of which are the reperfusion injury salvage kinase (RISK) signaling pathway that is an important mechanism against myocardial ischemia/reperfusion injury (Hausenloy and Yellon, 2007). PI3K and ERK1/2 phosphorylation are involved in ischemic preconditioning and drug-mediated anti-myocardial ischemia/reperfusion injury process. Activation of PI3K can promote the phosphorylation of endothelial nitric oxide synthase (eNOS), p70S6K, GSK-3β, thereby inhibiting mPTP and playing a protective role (Gu et al., 2018); ERK1/2 signaling pathway activation can inhibit the activation of protease caspase-3, thereby inhibiting cardiomyocyte apoptosis. The activation of both signaling pathways can inhibit the expression of pro-apoptotic proteins Bim, Bax, Bad, thus preventing ischemic cardiomyocytes from necrosis or apoptosis (Hausenloy et al., 2005). Thus, we inferred that MS against myocardial ischemia/reperfusion injury via RISK signaling pathway through inhibiting circ003593.

In light with recent results, we will investigate the expressions of circ_003593 in human samples and validate the network of circRNA-targeted miRNAs and mRNAs in further studies. This study is the first time to describe the protective role of MS on I/R injury. Our findings reveal a novel circRNA circ_003593 that regulates cardiomyocyte apoptosis and proliferation and may serve as a potential therapeutic target for ischemic heart diseases.

## Data Availability Statement

The datasets generated for this study are available on request to the corresponding author.

## Ethics Statement

The animal study was reviewed and approved by the Ethical Committee for Animal Research of Central South University.

## Author Contributions

YX and YL conceived and designed the study and experiments. SW and DBO performed the experiments in vitro and analyzed the data. DBO provided the reagents and performed the animal experiments. All authors contributed to the article and approved the submitted version.

## Funding

This research was supported by Natural Science Foundation of Hunan Province (2018JJ3864 to SW).

## Conflict of Interest

The authors declare that the research was conducted in the absence of any commercial or financial relationships that could be construed as a potential conflict of interest.

## Supplementary Material

Supplementary Figure 1 The original Electrocardiogram (ECG) records. The ST changes in I/R rat model with or without MS pre-treatment. MS, *Malva sylvestris* L.; NS, normal saline; I/R, ischemia-reperfusion.
