# Supplementary figures and images for: Circular RNA Involved in the Protective Effect of Malva sylvestris L. on Myocardial Ischemic/Re-Perfused Injury
Source: Front Pharmacol. 2020 Sep 25;11:520486. doi: 10.3389/fphar.2020.520486 (PMC7546788; doi:10.3389/fphar.2020.520486)

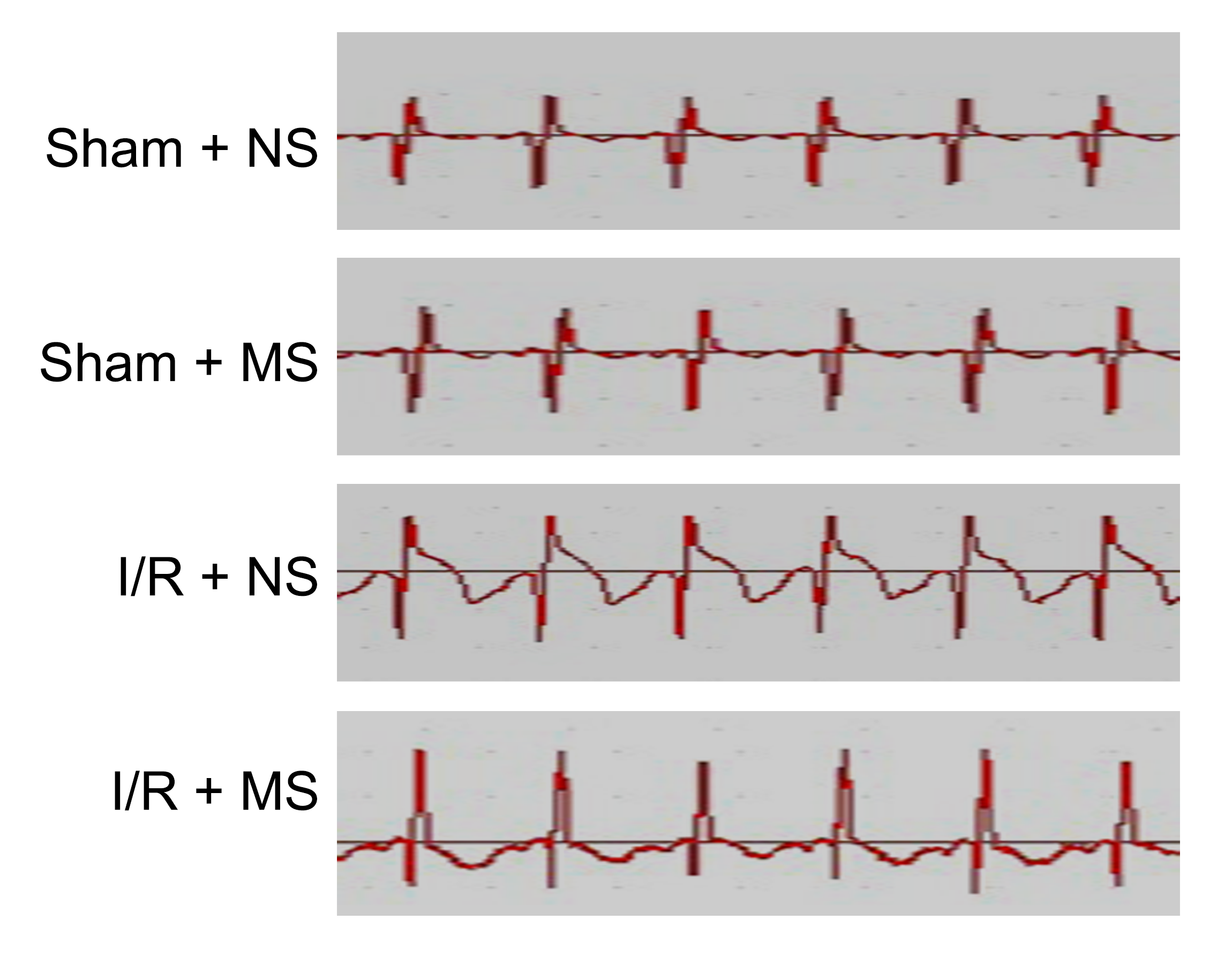

Supplement: Supplementary Figure 1 — The original Electrocardiogram (ECG) records. The ST changes in I/R rat model with or without MS pre-treatment. MS, Malva sylvestris L.; NS, normal saline; I/R, ischemia-reperfusion. [file Image_1.tif]
